# Changes in circulating 25-hydroxyvitamin D according to vitamin D binding protein genotypes after vitamin D_3_ or D_2_ supplementation

**DOI:** 10.1186/1475-2891-12-39

**Published:** 2013-04-04

**Authors:** Hataikarn Nimitphong, Sunee Saetung, Suwannee Chanprasertyotin, La-or Chailurkit, Boonsong Ongphiphadhanakul

**Affiliations:** 1 Division of Endocrinology and Metabolism, Department of Medicine, Faculty of Medicine, Ramathibodi Hospital, Mahidol University, Rama 6 Rd, Rajthevi, Bangkok 10400, Thailand

**Keywords:** 25-hydroxyvitamin D, Vitamin D_3_, Vitamin D_2_, Vitamin D binding protein(DBP)

## Abstract

**Background:**

It is not known whether genetic variation in the vitamin D binding protein (DBP) influences 25-hydroxyvitamin D levels [25(OH)D] after vitamin D supplementation. We aimed to investigate the changes of total 25(OH)D, 25(OH)D_3_ and 25(OH)D_2_ in a Thai cohort, according to type of vitamin D supplement (vitamin D_3_ or D_2_) and DBP genotype, after receiving vitamin D_3_ or D_2_ for 3 months.

**Methods:**

Thirty-nine healthy subjects completed the study. All subjects received 400 IU of either vitamin D_3_ or D_2_, plus a calcium supplement, every day for 3 months. Total serum 25(OH)D, 25(OH)D_3_ and 25(OH)D_2_ were measured by LC-MS/MS. Individual genotyping of rs4588 in the *DBP* gene was performed using real-time PCR.

**Results:**

Vitamin D_3_ supplementation of 400 IU/d increased 25(OH)D_3_ significantly (+16.2 ± 4.2 nmol/L, *p* <0.001). Vitamin D_2_ (400 IU/d) caused increased 25(OH)D_2_ levels (+22.0 ± 2.11 nmol/L, *p* <0.001), together with a decrease of 25(OH)D_3_ (−14.2 ± 2.0 nmol/L, *p* <0.001). At 3 month, subjects in vitamin D_3_ group tended to have higher total 25(OH)D levels than those in vitamin D_2_ (67.8 ± 3.9 vs. 61.0 ± 3.0 nmol/L; *p* = 0.08). Subjects were then classified into two subgroups: homozygous for the *DBP* rs4588 C allele (CC), and the rest (CA or AA). With D_3_ supplementation, subjects with CA or AA alleles had significantly less increase in 25(OH)D_3_ and total 25(OH)D when compared with those with the CC allele. However, no difference was found when the supplement was vitamin D_2_.

**Conclusion:**

Genetic variation in *DBP* (rs4588 SNP) influences responsiveness to vitamin D_3_ but not vitamin D_2_.

## Background

Vitamin D insufficiency has been increasingly recognized as a common health problem worldwide. Measures to augment vitamin D levels include increased sun exposure, higher consumption of vitamin D–rich foods, and taking vitamin D supplements. In order to achieve a 25-hydroxyvitamin D level [25(OH)D; a marker of vitamin D status] higher than the current recommended threshold of 50–75 nmol/L, higher doses of vitamin D than previously suggested are required in Caucasians [1,2]. However, it is unclear if the suggestion holds across ethnic groups, particularly in populations with lower body fat as compared to Caucasians. Moreover, it has never been investigated in Asians if vitamin D_2_ or D_3_ supplementation would have a different effect on circulating vitamin D.

Vitamin D and its metabolites circulate in the plasma, bound to vitamin D binding protein (DBP) [3]. Genetic variations in the *DBP* gene have consistently been found to be associated with 25(OH)D levels [4-7]. However, it is currently unclear how the *DBP* genetic variation would affect the increase in 25(OH)D levels after taking vitamin D supplements.

In the present study, we investigated the changes of total 25(OH)D, 25(OH)D_3_ and 25(OH)D_2_ according to type of vitamin D supplement (400 IU of vitamin D_3_ or D_2_ for 3 months). Toward the end, we investigated the change in serum 25(OH)D levels according to *DBP* genotypes.

## Methods

### Subjects

A total of 41 subjects (34 females/7 males) aged 15–70 years were enrolled in an unblinded randomized control trial that began in August 2008. All subjects were healthy and did not have any underlying diseases. Subjects were excluded if they were taking vitamin D ≥400 IU/d before being included in the study. After inclusion, subjects were excluded from the final analysis if they were intolerant to the supplement or were lost to follow-up. Subjects were then randomly assigned into two groups, using a computer-generated randomized code, to receive 400 IU/day of either vitamin D_2_ or vitamin D_3_ for 3 months. Two subjects (both of them were females) in the vitamin D_2_ group were excluded from the study after enrollment; one subject had gastrointestinal side effects from the calcium supplement, and the second was lost to follow-up. Final analysis was based on data from 39 subjects (32 females/7 males): 20 and 19 subjects in the vitamin D_3_ and D_2_ groups, respectively. All subjects were supplemented during the rainy or winter season and we did not allow taking any additional vitamin supplement. The treatment period was only 3 months and all subjects stayed in the same environment. In addition, there is less natural vitamin D containing food and vitamin D fortified food in Thailand. Therefore we did not assess the duration of sun exposure and the amount of daily vitamin D intake.

All study participants arrived at the research unit at 0800 h after at least a 12 h overnight fast. Baseline characteristics – which included age, all medications currently in use, waist circumference (WC) and body mass index (BMI) – were recorded. Blood was collected at baseline. Subjects received either vitamin D_3_ (Centrum^®^) or vitamin D_2_ (MTV), as well as a calcium supplement (CaCO_3_) every day, and were asked to return to the clinic at 1 month and 3 months after the first visit. Fasting plasma glucose (FPG) was measured at baseline, and total serum 25(OH)D, 25(OH)D_2_ and 25(OH)D_3_ were measured at baseline and at every follow-up period. Intact plasma parathyroid hormone (PTH) was measured at baseline and 3 month. Subjects with total 25(OH)D levels <50 nmoL/L were classified as having vitamin D deficiency. 25(OH)D, 25(OH)D_2_ and 25(OH)D_3_ were reported as levels (nmol/L) and as changes from baseline over the follow-up period (1 and 3 month).

### Vitamin D and calcium supplementation

All subjects received either vitamin D_3_ or vitamin D_2_ and a calcium supplement daily for 3 months. Subjects in the vitamin D_3_ group received 400 IU/d vitamin D_3_ and 675 mg/d elemental calcium [1 tablet of Centrum^®^ (contained 400 IU of vitamin D_3_ and 175 mg of elemental calcium) and 1 tablet of 1250 mg CaCO_3_ (contained 500 mg of elemental calcium)]; subjects in the vitamin D_2_ group received 400 IU/d vitamin D_2_ and 500 mg/d elemental calcium [1 multivitamin tablet (contained 400 IU of vitamin D_2_) and 1 tablet of 1250 mg CaCO_3_ (contained 500 mg of elemental calcium)]. Compliance was assessed by tablet-counting at every return visit, and was reported as % of medicine taken. All subjects had over 90% compliance for calcium, vitamin D_2_ and vitamin D_3_.

### Biochemical measurement

Serum and plasma samples were kept frozen at −80°C until analysis. Plasma intact parathyroid hormone (PTH) was determined by electrochemiluminescence immunoassay with an Elecsys 2010 analyzer (Roche Diagnostics, Mannheim, Germany). Serum 25(OH)D_2_ and 25(OH)D_3_ were analyzed by LC-MS/MS with an Agilent 1200 Infinity liquid chromatograph (Agilent Technologies, Waldbronn, Germany) coupled to a QTRAP^®^ 5500 tandem mass spectrometer (AB SCIEX, Foster City, CA, USA) using a MassChrom^®^ 25-OH-Vitamin D_3_/D_2_ diagnostics kit (ChromSystems, Munich, Germany). The summation of serum 25(OH)D_2_ and 25(OH)D_3_ was used to reflect vitamin D status. The inter-assay and intra-assay coefficients of variation of serum total 25(OH)D level were 6.3% and 5.0%, respectively.

### SNP genotyping

DNA was extracted from a 200 μL serum sample using a QIAamp^®^ DNA Blood Mini Kit (Qiagen, Hilden, Germany) according to the manufacturer’s protocol. Individual genotyping of rs4588 in the *DBP* gene was performed using real-time PCR (TaqMan^®^ MGB probes): 20 ng of DNA was added into the PCR reaction, consisting of TaqMan Universal Master Mix (1×) and TaqMan MGB probes for intronic C/A SNP rs4588 (1×) in a total volume of 20 μL. The real-time PCR reaction protocol was 10 min at 95°C, 40 cycles of 15 s at 92°C, and 1 min at 60°C using a 7500 Real-Time PCR System (Applied Biosystems, Foster City, CA, USA).

### Statistical analysis

Data were expressed as mean ± SEM unless stated otherwise. We performed non-parametric test to compare the difference between parameters. We used the Mann–Whitney test and the Chi-Square test to compare the difference of baseline characteristics and changes in vitamin D metabolites [total 25(OH)D, 25(OH)D_2_ and 25(OH)D_3_] between groups of subjects stratified by vitamin D preparations and DBP genotypes. The changes of vitamin D metabolites at the 1^st^ and 3^rd^ month from baseline in each group of subjects were assessed by the Wilcoxon test. All statistical analyses were performed with SPSS version 16 (SPSS, Chicago, IL). A *P* value of <0.05 was considered statistically significant.

## Results

**Figure 1:**
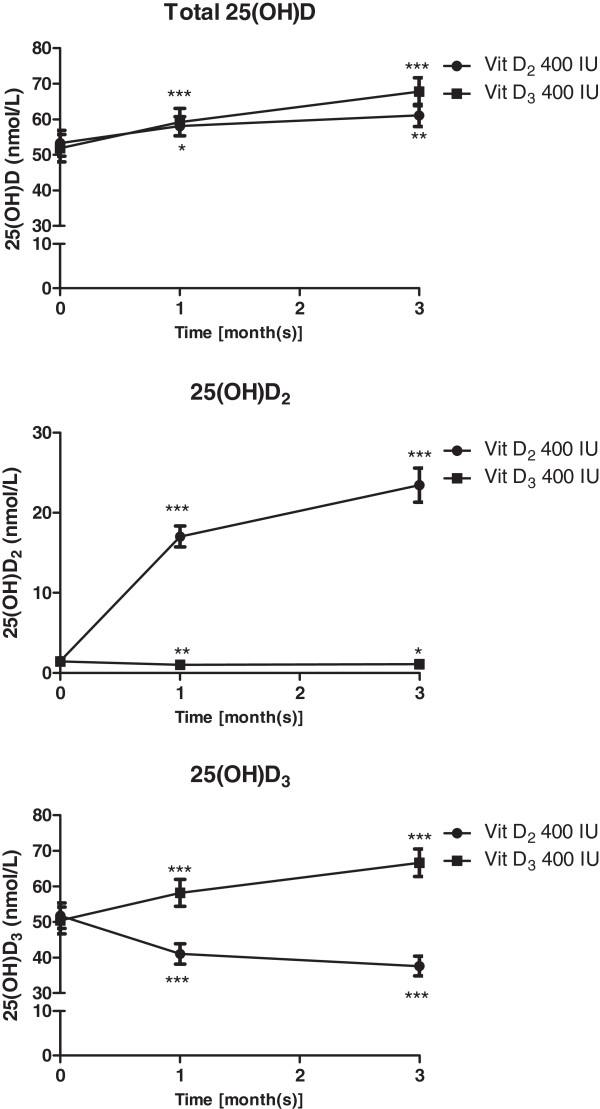
**Total 25(OH)D, 25(OH)D**_**2 **_**and 25(OH)D**_**3 **_**levels at baseline, and at 1 and 3 months after vitamin D**_**3 **_**or vitamin D**_**2 **_**supplementation.**

**Figure 2:**
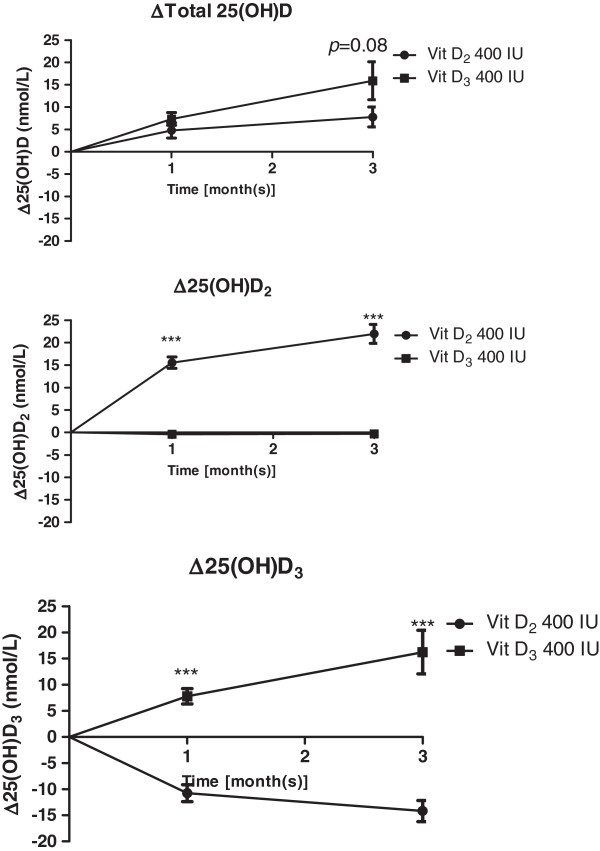
**The differences of total 25(OH)D, 25(OH)D**_**2 **_**and 25(OH)D**_**3 **_**levels from baseline at 1 and 3 months after vitamin D**_**3 **_**or vitamin D**_**2 **_**supplementation.**

Thirty-nine subjects (82.05% female) with a mean age of 36.2 ± 1.3 were included in the final analysis. All baseline characteristics of subjects in both vitamin D_3_ and vitamin D_2_ groups were similar (Table 1). Twenty-four out of 39 subjects (61.5%) had vitamin D deficiency [25(OH)D less than 50 nmol/L]: 14 and 10 subjects in the vitamin D_3_ and vitamin D_2_ groups, respectively (*p* = NS). As shown in Figures 1 and 2, after receiving 400 IU/d vitamin D_3_, 25(OH)D_3_ levels increased significantly (7.8 ± 1.5 and 16.2 ± 4.2 nmol/L at the 1^st^ and the 3^rd^ month, respectively; *p* < 0.001), with a small concurrent decrease in 25(OH)D_2_. Among subjects who received 400 IU/d vitamin D_2_, serum 25(OH)D_2_ levels increased by 15.6 ± 1.3 and 22.0 ± 2.1 nmol/L at the 1^st^ and 3^rd^ month, respectively; there was, however, a significant decrease of serum 25(OH)D_3_ (−14.2 ± 2.0 nmol/L at the 3^rd^ month; *p* < 0.001). This resulted in a reduction in the increase of total 25(OH)D levels after vitamin D_2_ supplementation. Nevertheless, when compared between the vitamin D_2_ and D_3_ groups, there were no significant difference in total 25(OH)D levels at 3 months from baseline [total 25(OH)D = 61 ± 3.1 vs. 67.6 ± 3.9 nmol/L and the increased of total 25(OH)D from baseline = 7.8 ± 2.2 vs. 15.9 ± 4.3 nmol/L (*p* = 0.08) for vitamin D_2_ and vitamin D_3_ group, respectively; Figure 1A). At the 3^rd^ month, plasma intact PTH was decreased significantly from baseline in subjects of both vitamin D_2_ [−0.6 ± 0.3 pmol/L, *p* < 0.05] and D_3_ group [−0.7 ± 0.2 pmol/L, p < 0.01]. There was no difference in the decrement in intact PTH between 2 groups (*p* = 0.7).

**Table 1:** Baseline characteristics according to vitamin D supplementation

**Clinical characteristics**	**Vitamin D**_**3 **_**(n = 20)**	**Vitamin D**_**2 **_**(n = 19)**	***P *value**
Age (years)	36.0 ± 1.9	36.7 ± 1.7	NS
Sex (M/F)	3/17	4/15	NS
Body weight (kg)	56.9 ± 2.0	59.5 ± 2.7	NS
Waist circumference (cm)	75.9 ± 1.5	78.4 ± 2.1	NS
BMI (kg/m^2^)	22.4 ± 0.5	23.2 ± 0.8	NS
FPG (mmol/L)	4.6 ± 0.1	4.5 ± 0.1	NS
Total 25(OH)D (nmol/L)	51.8 ± 3.8	53.2 ± 3.6	NS
25(OH)D_3_ (nmol/L)	50.3 ± 3.8	51.7 ± 3.6	NS
25(OH)D_2_ (nmol/L)	1.5 ± 0.1	1.5 ± 0.2	NS
PTH (pmol/L)	3.9 ± 0.3	4.0 ± 0.4	NS

Data is presented as mean ± SEM.

Table 2 shows the genotype distribution of the *DBP* rs4588 SNP. The genotype distributions of subjects given vitamin D_3_ or vitamin D_2_ supplements were not significantly different. Subjects in each vitamin D supplementation group were then divided into two subgroups based on the presence of the A (minor) allele: those homozygous for the C allele (group 1), and the rest (CA or AA: group 2). When we compared baseline 25(OH)D and 25(OH)D_3_ levels in subjects between group 1 (n = 22) and group 2 (n = 17), there was no difference in either baseline 25(OH)D (52.9 ± 4 vs. 52.1 ± 3 nmol/L, *p* = 0.5) or baseline 25(OH)D_3_ levels (51.4 ± 4.1 vs. 50.6 ± 3 nmol/L, p = 0.6). We further classified subjects into 4 groups according to *DBP* genotype and type of vitamin D supplement (Table 3). While there was no difference in age, gender, BMI, and baseline 25(OH)D_2_, 25(OH)D_3_ and total 25(OH)D between the genotypes among subjects in the vitamin D_2_ group, some differences were found in gender, baseline 25(OH)D_3_ and total 25(OH)D between the genotypes among subjects in the vitamin D_3_ group (Table 3). When comparing changes in vitamin D metabolites at 3 months with baseline values (Table 4), it was found that subjects in group 2 (CA or AA alleles) had significantly less increase in 25(OH)D_3_ levels after taking vitamin D_3_. Likewise, the increment in total 25(OH)D was lower. On the other hand, no difference in the increments of 25(OH)D_3_, 25(OH)D_2_ or total 25(OH)D was detected between the two genotype groups when the supplement was vitamin D_2_. There was no difference in changes in serum intact PTH between two *DBP* genotype subgroups after 3 months of vitamin D supplementation (Table 4).

**Table 2:** **Genotype distribution of the *DBP *rs4588 SNP in the study population**

**DBP genotype**	**D**_**3 **_**supplement (n = 20)**	**D**_**2 **_**supplement (n = 19)**	**Total**
CC (%)	12 (60%)	10 (52.6%)	22 (56.4%)
CA (%)	6 (30%)	5 (26.3%)	11 (28.2%)
AA (%)	2 (10%)	4 (21.1%)	6 (15.4%)

**Table 3:** **Baseline characteristics according to *DBP *genotype and type of vitamin D supplement**

	**D**_**3 **_**supplement**			**D**_**2 **_**supplement**		
	**CC (n = 12)**	**CA/AA (n = 8)**	***P *value**	**CC (n = 10)**	**CA/AA (n = 9)**	***P *value**
Age (years)	37.6 ± 2.6	33.5 ± 2.8	NS	36.1 ± 2.1	37.4 ± 2.8	NS
% female	100	62.5	<0.05	72.7	88.9	NS
BMI (kg/m^2^)	22.0 ± 0.7	22.9 ± 0.9	NS	23.6 ± 1.1	22.7 ± 1.2	NS
25(OH)D_3_ (nmol/L)	47.4 ± 5.7	55.0 ± 4.2	<0.05	56.3 ± 5.7	46.8 ± 3.9	NS
25(OH)D_2_ (nmol/L)	1.6 ± 0.3	1.3 ± 0.1	NS	1.4 ± 0.2	1.6 ± 0.3	NS
total 25(OH)D (nmol/L)	49.0 ± 5.8	56.3 ± 4.2	<0.05	57.7 ± 5.7	48.3 ± 3.9	NS
PTH (pmol/L)	4.0 ± 0.5	3.7 ± 0.5	NS	4.0 ± 0.6	3.9 ± 0.6	NS

Data is presented as mean ± SEM.

**Table 4:** **Changes in vitamin D metabolites and PTH at 3 months compared to baseline according to *DBP *genotypes and vitamin D preparations**

	**D**_**3 **_**supplement**			**D**_**2 **_**supplement**		
	**CC (n = 12)**	**CA/AA (n = 8)**	***P *value**	**CC (n = 10)**	**CA/AA (n = 9)**	***P *value**
Δ 25(OH)D_3_ (nmol/L)	22.98 ± 6.00	6.09 ± 3.03	<0.01	−15.38 ± 2.60	−12.85 ± 3.21	NS
Δ 25(OH)D_2_ (nmol/L)	−0.39 ± 0.36	−0.28 ± 0.10	NS	23.31 ± 3.59	20.47 ± 2.11	NS
Δ 25(OH)D (nmol/L)	22.58 ± 6.18	5.84 ± 3.07	<0.01	7.93 ± 3.10	7.61 ± 3.35	NS
Δ PTH (pmol/L)	−0.8 ± 0.3	−0.7 ± 0.4	NS	−0.6 ± 0.5	−0.7 ± 0.4	NS

Data is presented as mean ± SEM.

## Discussion

In the present study, 400 IU/day of vitamin D_3_ tended to increase total 25(OH)D levels more when compared with the same dosage of vitamin D_2_ (*p* = 0.08). The underlying basis for this finding appears to be a concurrent decrease in 25(OH)D_3_ after supplementation with vitamin D_2_. This finding is in keeping with a number of studies which demonstrated a decrease in 25(OH)D_3_ after vitamin D_2_ supplementation either daily or weekly [8-10]. However, there was also one other study which could not demonstrate a concurrent decrease in 25(OH)D_3,_ and found that vitamin D_2_ and D_3_ were equipotent in improving vitamin D status [11]. It is noteworthy that nearly all of the studies that demonstrated less efficacy of vitamin D_2_ in increasing 25(OH)D levels used relatively higher doses of vitamin D, indicating that the less efficacy of vitamin D_2_ may be dose-dependent [9,12]. The recent meta-analysis indicated that vitamin D3 is more efficacious at raising serum 25(OH)D concentration when given as a bolus dose [50,000 IU single dose (oral), 300,000 IU single dose (oral and intramuscular) and 50,000 IU/month (oral); *p* = 0.0002] compared with administration of vitamin D_2_, but the effect was lost with daily supplement [1,000-4,000 IU/d; *p* = 0.10] [13]. However, our findings suggest that this is not the case, since we were able to demonstrate the trend of the difference between D_3_ and D_2_ supplementation even at a lower dose, i.e. 400 IU daily. All of the previous studies were performed in Caucasians, and it is unclear if ethnic differences in vitamin D metabolism could be the basis of our findings. The decrease in 25(OH)D_3_ after vitamin D_2_ supplementation, same as reported in the study of Demetriou ET, et al. [14], likely due to competition for the 25-hydroxylase enzyme by vitamin D_3_ and vitamin D_2_. However, it is probable that enzymatic catalyzation by other enzymes with relatively minor roles, such as CYP24A1 [8] and CYP3A4, may be different for vitamin D_3_ and D_2_, and thus be partially accountable for the observation.

The ability of either vitamin D_2_ or vitamin D_3_ to increase circulating 25(OH)D varies considerably among individuals. The explanations for the large between-individual difference include differences in adiposity [15], enzymatic degradation of vitamin D metabolites [8], and dietary composition [16], as well as fat malabsorption [17]. In the present study, vitamin D_2_ or vitamin D_3_ supplementation resulted in varying increases in 25(OH)D. No association with adiposity as assessed by BMI, however, was demonstrated. Since the number of study subjects was small, the lack of power to detect association could possibly be responsible. Dietary composition and enzymatic degradation of vitamin D metabolites were not assessed in the present study.

The present study demonstrated that DBP genetic variation is another factor which can influence the responsiveness to vitamin D supplementation. The major function of DBP is the binding, solubilization and transport of vitamin D and its metabolites [18]. A previous study found that both serum 25(OH)D_3_ and 1, 25(OH)_2_D_3_ concentrations were significantly lower in mice lacking DBP, compared to wild-type mice [19]. In another recent study, Lauridsen *et al*. showed that DBP phenotype determines the median plasma concentration of 25(OH)D_3_ and 1, 25(OH)_2_D_3_[5]. With regard to genetic variation, single nucleotide polymorphisms in the *DBP* gene have been demonstrated to be related to 25(OH)D levels in Caucasians and Africans [4,6]. A difference in the response of serum 25(OH)D after vitamin D_3_ supplementation according to DBP genetic variants has also been reported [7]. It is of note that the DBP genetic variants affected the change in vitamin D status only for vitamin D_3_ but not vitamin D_2_ supplementation in our study. One study reported that vitamin D_3_ had greater affinity for the DBP than vitamin D_2_ and 25(OH)D_3_ also had greater affinity for the DBP than 25(OH)D_2_[20]. However, the binding capacity of DBP cannot totally explain the difference in responsiveness to vitamin D_3_ supplementation as demonstrated in Armas, LA.’s study [9]. After receiving single dose of 50,000 vitamin D_3_ or vitamin D_2_ orally, a similar rising of 25(OH)D levels in the first 3 days were observed in both group. However, much more rapid decline of serum 25(OH)D in the vitamin D_2_-treated subjects after 3 days seem to reflect substantially more rapid metabolism or clearance of the vitamin D_2_ metabolite [9]. We speculated that the greater affinity of 25(OH)D_3_ for the DBP and less clearance of 25(OH)D_3_ would maintain its serum levels and accountable for the influence of DBP genetic variants in the case of vitamin D_3_,not vitamin D_2_. Further studies such as the differences in metabolism between vitamin D_2_ and vitamin D_3_ after binding to DBP are warranted.

There are a number of limitations in this study. The sample size is relatively small and may not have enough power to detect small changes, particularly those related to vitamin D_2_ supplementation. A 50 nmol/L difference in baseline 25(OH)D_2_ and 25(OH)D_3_ was present. This may influence the increment of 25(OH)D_2_ or 25(OH)D_3_ levels after receiving vitamin D_2_ or vitamin D_3_ supplementation, and thus possibly affect the total 25(OH)D levels at the end of the study. The change in 25(OH)D at the end of the study may also be influenced by lifestyle factors influencing the degree of sun exposure during the course of the study. This potential confounding effect cannot be assessed since there was not a non-supplemented group in our study. It would be important to know if both the vitamin D_2_ and vitamin D_3_ supplements had the same amount of vitamin D. However, vitamin D contents of both preparations were not measured. However, total 25(OH)D levels of subjects in both groups were similar at 3 months, suggesting that the variability in vitamin D content between vitamin D_2_ and vitamin D_3_ preparations, if any existed, was likely to be small. The other limitation is that multivitamin tablets containing vitamin D were used (in the vitamin D_2_ group), and it is unknown if other constituents of the multivitamin would affect vitamin D absorption or metabolism. It has been demonstrated that statins, atorvastatin and rosuvastatin in particular, can influence the circulating levels of 25(OH)D [21-23]. To our knowledge, no interference from common vitamins and minerals with vitamin D metabolism has been reported.

## Conclusion

Daily supplementation with vitamin D_2_ tended to result in lower total 25(OH)D levels than supplementation with vitamin D_3_ due to a concurrent decrease in 25(OH)D_3_ levels in the former case. Genetic variation in vitamin D binding protein (rs4588 SNP) influences responsiveness to vitamin D_3_ but not vitamin D_2_.

## Competing interest

The authors have nothing to declare.

## Authors’ contribution

HN and BO conceived of the study, participated in its design and coordination, performed the statistical analysis and helped to draft the manuscript. SS carried out the genotyping of rs4588 in the *DBP* gene. SC carried out the biochemical measurement. LC carried out the vitamin D metabolites measurement (LC-MS/MS). All authors read and approved the final manuscript.
